# Crystallization-Controlled Structure and Thermal Properties of Biobased Poly(Ethylene2,5-Furandicarboxylate)

**DOI:** 10.3390/polym16213052

**Published:** 2024-10-30

**Authors:** Miroslaw Pluta, Joanna Bojda, Mariia Svyntkivska, Tomasz Makowski, Ele L. de Boer, Ewa Piorkowska

**Affiliations:** 1 Centre of Molecular and Macromolecular Studies, Polish Academy of Sciences, Sienkiewicza 112, 90-363 Lodz, Poland; joanna.bojda@cbmm.lodz.pl (J.B.); mariia.svyntkivska@cbmm.lodz.pl (M.S.); ewa.piorkowska@cbmm.lodz.pl (E.P.); 2 Avantium Renewable Polymers BV, Zekeringstraat 29, 1014 BV Amsterdam, The Netherlands; ele.deboer@avantium.com

**Keywords:** poly(ethylene 2,5-furandicarboxylate), cold crystallization, polymorphism, morphology

## Abstract

Crystallization-controlled structure and thermal properties of biobased poly(ethylene 2,5-furandicarboxylate) (PEF) were studied. The cold-crystallization temperature controlled the structure and thermal properties of the biobased PEF. The melting was complex and evidenced the presence of a significant fraction of less-stable crystals with a low melting temperature that linearly increased with *T*_c_, which formed already during the early stages of crystallization, together with those melting at a higher temperature. Low *T*_c_ resulted in the α’-phase formation, less crystallinity, and greater content of the rigid amorphous phase. At high *T*_c_, the α-phase formed, higher crystallinity developed, the rigid amorphous phase content was lower, and the melting temperature of the less-stable crystals was higher; however, slight polymer degradation could have occurred. The applied thermal treatment altered the thermal behavior of PEF by shifting the melting of the less stable crystals to a significantly higher temperature. SEM examination revealed a spherulitic morphology. A lamellar order was evidenced with an average long period and small average lamella thickness, the latter about 3–3.5 nm, only slightly increasing with *T*_c_.

## 1. Introduction

Poly(ethylene 2,5-furandicarboxylate) (PEF), a thermoplastic polyester, is produced most often from monomers: 2,5-furandicarboxylic acid and mono-ethylene glycol [1,2,3,4] or less frequently by ring-opening polymerization of cyclic PEF oligomers [5,6]. Although PEF has been known for nearly 80 years [7], recently it has gained a growing interest owing to its physical properties and the development of new technologies enabling large-scale production from bio-based substrates [8]. PEF is a potential biobased replacement of widely used poly(ethylene terephthalate) (PET), especially since it offers improved mechanical and gas barrier properties compared to PET and other petrol-based terephthalate homologs. Moreover, PEF is recyclable using technology developed for PET. Interestingly, in mechanical recycling, up to 5% of PEF can be processed together with PET without the deteriorated performance of the recycled polymer [9].

PEF is an analog to PET, in which a benzene ring is replaced with a furan ring, as shown in Figure S1 in SM (Supplementary Materials). A consequence of the absence of furan ring flipping and the lower bond angle of the carboxyl groups on the ring, as compared to the arrangement on the benzene ring, is a higher energy barrier for cooperative motions, which results in decreased PEF chain mobility in the amorphous state [10]. This difference is a reason for improved PEF mechanical and gas barrier properties compared to PET and other petrol-based terephthalate homologs. For example, PEF exhibits a higher elastic modulus and tensile strength [11,12,13], a higher barrier for oxygen [14], carbon dioxide [15], and water vapor [16]. Moreover, it can be melt processed at a lower temperature than PET. Recently, PEF fibers were electrospun [17] and did not exhibit any toxic effect on healthy human keratinocytes [18].

The supramolecular structure of PEF depends on its thermal history. PEF exhibits polymorphism and can crystallize in three different crystallographic modifications. At high temperatures, PEF crystallizes in the more ordered α-form, while at low temperatures in the less ordered α’-form, which can reorganize upon further heating [3,19,20,21,22,23,24]. Moreover, PEF crystallizes in the β-phase from solutions [25]. Recently, Maini et al. [23] proposed for PEF a triclinic unit cell for the α’-form and monoclinic unit cells for the α- and β-forms, based on X-ray diffraction powder patterns. PEF can be quenched to the glassy amorphous state without crystallization and cold-crystallized upon subsequent heating.

To date, little research has been conducted on the morphology of PEF in contrast to PET. Thus, the main aim of this work was to study systematically the supramolecular structure of biobased PEF, its crystal structure, and thermal behavior, depending on cold-crystallization temperature. Cold-crystallization was selected as such crystallization is faster than that after cooling the melt, and it was reported previously that both procedures lead to similar crystal structures [20,26]. To examine the internal structure, fracture surfaces were analyzed with scanning electron microscopy (SEM) after appropriate etching, and thin films were studied with polarized light microscopy (PLM). The structure was probed by wide-angle and small-angle X-ray scattering (WAXS and SAXS) to determine the crystallographic modifications and to gain insight into the lamellar organization. Differential scanning calorimetry (DSC) and thermogravimetry (TGA) enabled us to examine PEF thermal properties and the melting behavior depending on its thermal history.

## 2. Experimental

### 2.1. Materials

Poly(ethylene 2,5-furandicarboxylate) (PEF) was obtained in the form of granules from Avantium Renewable Polymers (Amsterdam, the Netherlands). The PEF grade was G90P with an intrinsic viscosity ([*η*]) of 0.86 dL/g, as measured according to ASTM D4603 [27], a weight-average molar mass (*M*_w_) of 128 kg/mol, and a polydispersity index (*PDI*) of 2.5 as determined using gel permeation chromatography with 1,1,1,3,3,3-hexafluoro-2-propanol as a solvent, and a classical calibration with a poly(methylene methacrylate) standard. The biobased PEF was synthesized from 2,5-furandicarboxylic acid obtained by catalytical conversion of plant-based sugars and from commercially available biobased mono-ethylene glycol. Figure S2 in SM shows the attenuated total reflectance Fourier transform infrared (ATR-FTIR) spectrum of amorphous PEF recorded with a Thermo Scientific Nicolet 6700 FT-IR instrument (Waltham, MA, USA), with a cm^−1^ resolution. The spectrum is typical of PEF with small peaks near 3000 cm^−1^ and a series of pronounced peaks below 1750 cm^−1^ [28,29].

### 2.2. Preparation

Before melt processing, PEF granules were dried overnight in a vacuum oven at 140 °C to reduce residual moisture content. Initial samples in the form of 0.2 mm thick sheets, qPEF, were obtained by compression molding of PEF pellets at 250 °C for 5 min in a laboratory press, followed by quick cooling down between aluminum blocks kept at room temperature (RT). At 250 °C the polymer processing was easier than at lower temperatures, 230–240 °C. To prevent sticking to the blocks, the specimens were sandwiched between 0.25 mm thick poly(tetrafluoroethylene) (PTFE) films (Polyflon Technology Ltd., Stafford, UK). Before the use, the PTFE films were cleaned with isopropanol and dried at 250 °C to evaporate it.

### 2.3. Crystallization

**Figure 1:**
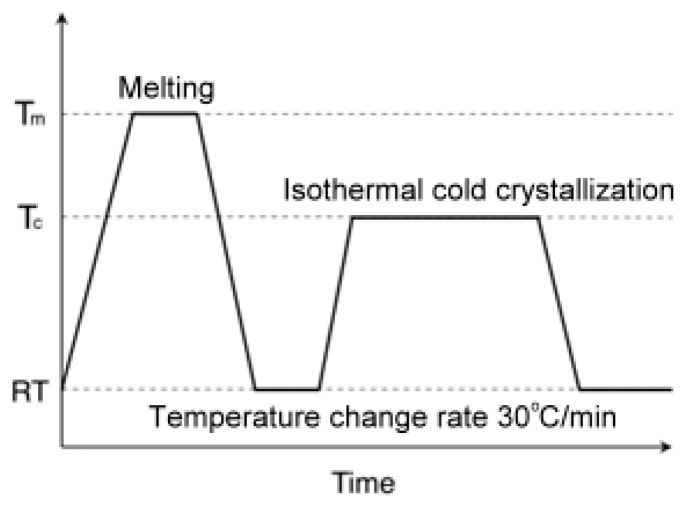
Temperature protocol for isothermal cold-crystallization of PEF at various temperatures, *T_c_*.

qPEF specimens, cut from the 0.2 mm thick sheets, were isothermally cold-crystallized in a Linkam CSS450 Optical Shearing System (Linkam, Waterfield, UK), between microscope slides, according to the temperature protocol shown in Figure 1. The cold-crystallization method was chosen because such crystallization is faster than the crystallization after cooling from above the melting temperature, most probably due to more intense primary nucleation. It is worth mentioning that both crystallization processes lead to similar crystal structures, as observed previously by others [20,26]. To prevent sticking to the microscope slides, the specimens were sandwiched between 0.125 mm thick Kapton 500 HN films (Micel, Saint-Chamond, France). The specimens were heated from RT to 250 °C at 30 °C/min and held at this temperature for 2 min to erase their thermal history, then cooled at 30 °C/min to 10 °C. After approx. 3 min, they were heated again at 30 °C/min to the selected crystallization temperatures (*T*_c_) ranging from 100 °C to 180 °C and held at these temperatures for 12–24 h to cold-crystallize them isothermally. The specimens were denoted as, for example, C120, where the number means *T*_c_. After 12–24 h, the specimens were cooled to 10 °C at 30 °C/min, removed from the hot stage, and then stored at RT. The times required for accomplishment of crystallization at *T*_c_ were determined based on preliminary DSC studies described in SM and Figure S3 in SM.

Also, 70 µm thick specimens, sandwiched between microscope cover slides, with the same thermal history as the thicker ones, were prepared for polarized light microscopy (PLM).

### 2.4. Characterization

The thermal properties of the PEF specimens were determined using DSC Q20 (TA Instruments, New Castle, DE, USA) during heating at 10 °C/min and 30 °C/min under a flow of nitrogen. In addition, the specimens cold-crystallized at *T*_c_ of 130–160 °C were re-heated in DSC at 10 °C/min to selected temperatures *T*_a_ above the first melting peak, held at these temperatures for 15–30 s, and cooled to RT at 30 °C/min. Then, the specimens were heated again from RT to 250 °C at 10 °C/min to analyze their melting behavior. These specimens are denoted with the letter a, for instance, C130a. In addition, C130a* was kept at *T*_a_ for 1 h.

Selected samples were analyzed with thermogravimetry using a TGA 5500 (TA Instruments) during heating from RT to 800 °C at a rate of 20 °C/min in both air and a nitrogen atmosphere. Temperatures at 5% mass loss (*T*_5%_) were determined based on the TGA thermograms, whereas temperatures of maximum decomposition rate (*T*_d_) were evaluated based on derivatives of weight loss with respect to temperature (DTGA). 

The viscoelastic properties of selected samples were examined with a dynamic mechanical thermal analysis (DMTA). The measurements were carried out on 0.5 mm thick rectangular specimens, 17.5 mm × 12 mm, in a single cantilever bending mode, using a DMTA TA Q-800 Thermal Analyser (TA Instruments) at a frequency of 1 Hz and a heating rate of 2 °C/min.

To determine their kinematic viscosity, the PEF specimens were dissolved at 90 °C in a mixture of phenol and 1,1,2,2-tetrachloroethane (6:4 *w:w*). The measurements of 20 mL portions of 1% PEF solutions were performed using a fully automated Herzog Multi-Range Viscometer HVM 472 at 25 °C. [*η*] was calculated using the Solomon–Ciutǎ equation [30,31]:

(1) $$\left[\eta \right]{=[2(t/t_{o}-ln(t/t_{o})-1)]}^{1/2}/c$$

where *c* is the concentration of the solution; *t* is the solution flow time; and *t*_o_ is the flow time of the pure solvent. For each specimen type, the measurements were repeated three times, and an average value was calculated.

WAXS was carried out in a reflection mode using an Aeris diffractometer (Malvern Panalytical Ltd., Malvern, UK) with CuKα radiation, λ = 0.154 nm, operating at 40 kV and 7.5 mA. The curves were recorded in a 2*θ* range from 5° to 50° with a step of 0.022° at RT. 

The lamellar structure was probed with a SAXS-2D, with a Kiessing-type camera, with a 1.2 m sample-detector distance, coupled to a low divergence X-ray CuKα micro-source operating at 50 kV and 1 mA, GeniX Cu- LD (Xenocs, Grenoble, France). The scattering patterns were recorded with a Pilatus 100K solid-state area detector with a resolution of 172 × 172 μm^2^ (Dectris, Baden, Switzerland) at RT. 

The structure was examined with a PLM Nikon Eclipse E400 (Nikon, Japan) equipped with a Sanyo VCC-3770P camera. Fracture surfaces of selected specimens were examined with a SEM JEOL 6010LA (Jeol, Tokyo, Japan), operating in a high vacuum mode at an accelerating voltage of 10 kV, after vacuum sputtering with a 10 nm gold layer using a coater Quorum EMS150R ES (Quorum Technologies, Lewes, UK). To better expose their internal structure, the specimens were etched according to the procedure used previously for polylactide (PLA) described in [32] and applied by others [33]. The specimens were immersed in a 0.05 M solution of sodium hydroxide in a mixture of methanol and distilled water (2:3 *v:v*) for 4 h at RT. Then, they were washed in an ultrasonic bath in a mixture of methanol and water for 5 min and dried.

## 3. Results and Discussion

### 3.1. Thermal Properties

**Figure 2:**
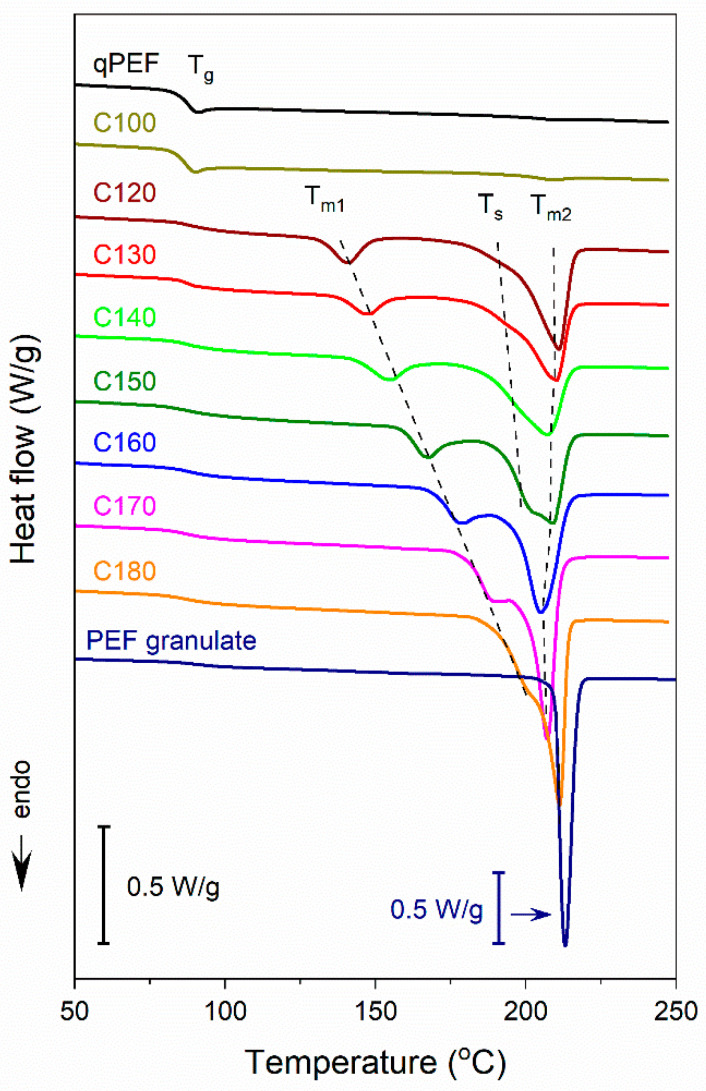
DSC heating thermograms of cold-crystallized PEF and PEF granulate recorded at 10 °C/min. Thermograms shifted vertically for clarity. Dashed lines indicate changes of melting temperatures (*T*_m1_, *T*_s_, and *T*_m2_) dependent on cold-crystallization temperature.

Figure 2 and Figure S4 show DSC heating thermograms of PEF specimens recorded on heating at 10 °C/min and 30 °C/min, respectively. The calorimetric data measured on heating at 10 °C/min are presented in Table 1. In the thermograms of qPEF recorded at 10 °C/min above the glass transition with *T*_g_ at 86.5 °C, only a very small cold-crystallization exotherm and a melting endotherm were observed. The cold-crystallization enthalpy (Δ*H*_cc_), below 0.5 J/g, was equal to the melting enthalpy (Δ*H*_m_), evidencing that qPEF was amorphous before the DSC experiment. C100 crystallized for 12h behaved similarly to qPEF, with *T*_g_ at 85.5 °C and a cold-crystallization peak temperature (*T*_cp_) of 182 °C, *T*_m_ of 209 °C, and Δ*H*_cc_ of 0.8 J/g equal to Δ*H*_m_. After 24 h at 100 °C, Δ*H*_cc_ and Δ*H*_m_ slightly increased to 1.3 J/g but were still equal, evidencing that even 24 h spent at 100 °C was insufficient to develop any detectable crystallinity at this temperature. During faster heating, at 30 °C/min, neither cold-crystallization nor melting occurred in qPEF (Figure S4). Crystallinity was detected only in PEF held at higher temperatures, well above 100 °C. 

All thermograms of these samples evidenced their complex melting behavior above the glass transition with *T*_g_ of 88–89 °C determined at 10 °C/min. At *T*_c_ of 120–160 °C, the main melting peaks with *T*_m2_ were accompanied by smaller low-temperature peaks with *T_m1_*. At *T*_c_ of 170 °C and 180 °C, these low-temperature melting peaks turned into shoulders of the main peaks. Moreover, during heating at 10 °C/min of PEF cold-crystallized at *T*_c_ of 130–150 °C, small shoulders appeared on the ascending slopes of the main peaks at temperatures *T*_s_. These shoulders were absent or hardly visible during heating at 30 °C/min (Figure S4). At *T*_c_ of 120 °C and 130 °C, during heating at 10 °C/min small exotherms were observed between the two melting peaks, with small enthalpies of 1.5 J/g and 0.2 J/g, respectively.

**Figure 3:**
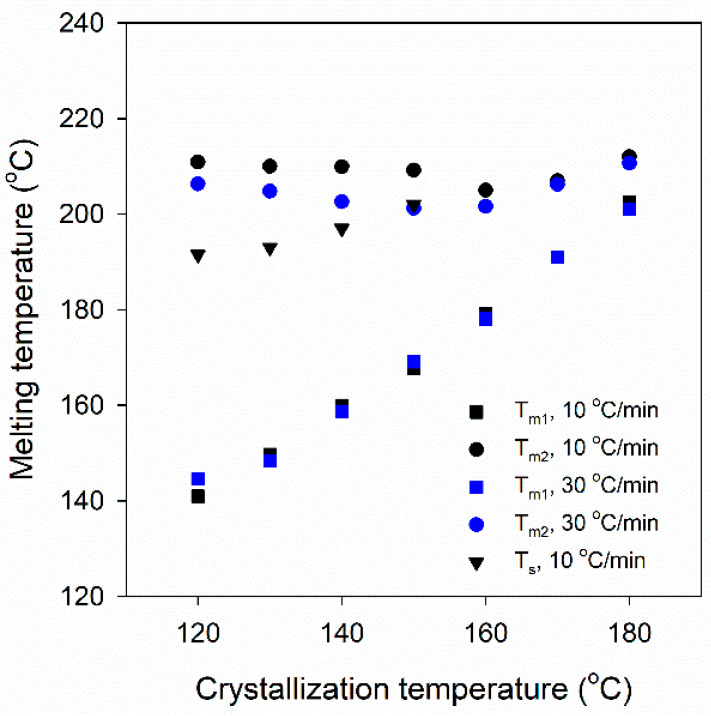
Melting temperatures, *T*_m1_, *T*_m2_, and *T*_s,_ of isothermally cold-crystallized PEF samples vs. crystallization temperature, *T*_c_.

Plots of *T*_m1_ and *T*_m2_ of the melting peaks and *T*_s_ of the small shoulders vs. *T*_c_ are shown in Figure 3. The values of *T*_m1_ were similar at both heating rates and increased linearly with the increasing *T*_c_. *T*_s_ also increased linearly with increasing *T*_c_. On the contrary, *T*_m2_ values were weakly dependent on *T*_c_, and they increased with increasing *T*_c_ only in the *T*_c_ range from 160 °C to 180 °C. Moreover, for *T*_c_ ≤ 160 °C, *T*_m2_ values were lower at 30 °C/min. These results indicate the occurrence of reorganization in the crystalline phase during heating, which was more intense during slower heating and caused an increase in *T*_m2_. 

**Figure 4:**
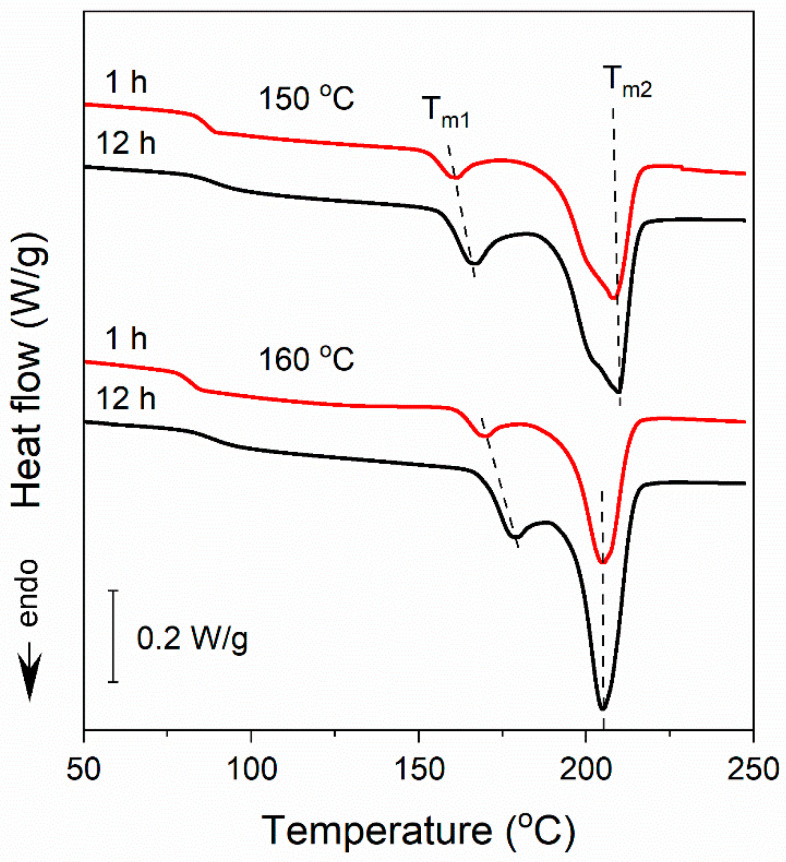
Comparison of heating thermograms, recorded at 10 °C/min, of specimens isothermally crystallized at temperature *T_c_* of 150 °C and 160 °C for 1 h and 12 h. Thermograms shifted vertically for clarity.

The crystallinity degree (*X_c_*), calculated based on the Δ*H*_mc_ measured at 10 °C/min, enlarged from approx. 30% to 40% with *T*_c_ increasing from 120 °C to 180 °C. At 10 °C/min and 30 °C/min, *T*_m1_ exceeded *T*_c_ by approx. 20 °C. As proposed by Papageorgiou et al. [3] and Stocklet et al. [20], by analogy with PET, the low-temperature melting peaks can be attributed to the melting of secondary small and imperfect crystals formed during crystallization at *T*_c_. The content of these crystals in the crystalline phase calculated based on the melting enthalpy data in Table 1 did not exceed 16% for *T*_c_ ≤ 140 °C. For higher *T*_c_, it was larger, approx. 20%, but the separation of the melting peaks in these cases was only approximate. The heating thermograms of PEF held at 150 °C and 160 °C for 1 h and 12 h, respectively, shown in Figure 4, offer a more detailed insight into the formation of the less stable crystals. It appears that the crystals with low *T*_m1_ were formed already at the early stages of crystallization, and that occurred at *T*_c_ range of the α’-phase formation (*T*_c_ ≤ 150 °C) and at higher *T*_c_ at which the α-phase was formed (*T*_c_ ≥ 160 °C), as shown further. However, after 1 h crystallization, *T*_m1_ values were by approx. 8–9 °C lower than those after 12 h crystallization at the same temperatures, which indicates the occurrence of isothermal annealing of the less stable crystals at *T*_c_, perfectioning, and/or thickening. On the contrary, the crystallization time did not influence *T*_m2_.

**Figure 5:**
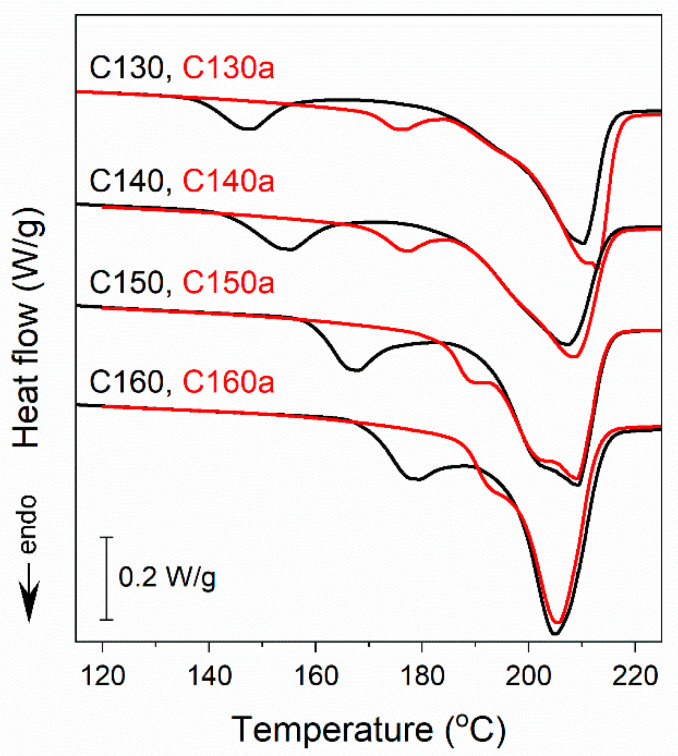
Comparison of DSC heating thermograms recorded at 10 °C/min of cold-crystallized C130, C140, C150, and C160 (black lines) and their re-heated counterparts C130a (*T*_a_ = 170 °C, *t*_a_ = 15 s), C140a (*T*_a_ = 172 °C, *t*_a_ = 30 s), C150a (*T*_a_ = 184 °C, *t*_a_ = 30 s), and C160a (*T*_a_ = 187 °C, *t*_a_ = 30 s) (red lines). Thermograms shifted vertically for clarity.

Figure 5 shows the heating thermograms of cold-crystallized PEF heated to temperatures *T*_a_, selected between the two melting peaks, near 170 °C for C130 and C140 and near 185 °C for C150 and C160, held there for 15–30 s and cooled quickly to RT at 30 °C/min. Although melting started at similar temperatures as during the first heating, the low-temperature melting peaks of C130 and C140 shifted to higher temperatures, whereas for C150 and C160 these peaks transformed into shoulders of the main peaks.

*T*_m1_ strongly increased, to 176 °C for C130 and C140 and to 191 °C and 194 °C for C150 and C160, respectively, whereas *T*_m2_ was nearly unaffected. It appears that during the treatment the less stable crystals melted and then recrystallized into more stable ones with higher *T*_m1_, which exceeded *T*_a_ by several degrees. In each case, these *T*_m1_ values were below those of PEF cold-crystallized at *T*_c_ close to *T*_a_. Taking into account the short dwell time at *T*_a_ and subsequent cooling in that temperature range where crystallization could occur, it could be concluded that the formation of the crystals was quite rapid, especially having in mind the long times required for the cold-crystallization of PEF. It is possible that this rapid crystallization was due to the melt memory.

Recently, it was assumed that the three-phase model is more suitable than the two-phase model for describing the structure of semi-crystalline polymers, including PEF [20], poly(butylene terephthalate) [34], PET [35], PTFE [36], and PLA [37]. The three-phase model takes into account the presence of a mobile amorphous fraction (*X*_maf_) being responsible for the glass-rubber transition and a rigid amorphous fraction (*X*_raf_), which is a constrained amorphous phase associated with the interface between the crystalline and the mobile amorphous phase. The relative fractions of these components can be estimated from calorimetric data according to the following relationships:

(2) $$X_{raf}=X_{a}-{\Delta C}_{p}/{\Delta C}_{p}^{a}$$

where *X*_a_ is the amorphous phase content, and Δ*C_p_* and Δ$C_{p}^{a}$ denote the heat capacity change due to the glass transition of a semicrystalline polymer and a fully amorphous polymer, respectively. Figure S5 shows the dependencies of *X*_c_, *X*_a_, and *X*_raf_ on *T*_c_ for the cold-crystallized PEF. It appears that *X*_raf_ decreased with the increasing *T*_c_, from 36% to 23% at 160 °C and then leveled off. *X*_am_ decreased with increasing *T*_c_, but the decrease in *X*_raf_ was stronger; *X*_raf_/*X*_a_ decreased from approx. 50% to 40%. RAF vitrification is connected to the mobility of the chains, which depends on *T*_c_. At higher temperatures, the polymer chains exhibit enhanced mobility, which facilitates the organization of the segments into ordered crystalline structures, with reduced stress transmitted to the amorphous segments and a lower fraction of chain segments subjected to geometrical constraints [38,39]. RAF affects polymer properties, for instance, the mechanical properties because it acts in the transmission of mechanical stress under load (affects the elastic modulus) [40], and the barrier properties as its free volume exceeds that of the MAF, which affects the diffusion of small molecules [41].

The viscoelastic properties of qPEF and C160 analyzed by DMTA are shown in Figure S6 in SM. The loss modulus (*E″*) curves exhibit low and broad peaks with maxima at −68 °C attributed to the relaxation related to carbonyl motions [10,42] and, in the glass transition region, peaks with maxima at 89 °C and 96 °C for qPEF and C160, respectively, differing because of the crystallinity of the latter. The storage moduli (*E’*) decreased with increasing temperature and dropped in the glass transition region, as observed by others [10,42]. *E’* of C160 exceeded that of qPEF in a broad temperature range and less sharply decreased in the glass transition region. 

**Figure 6:**
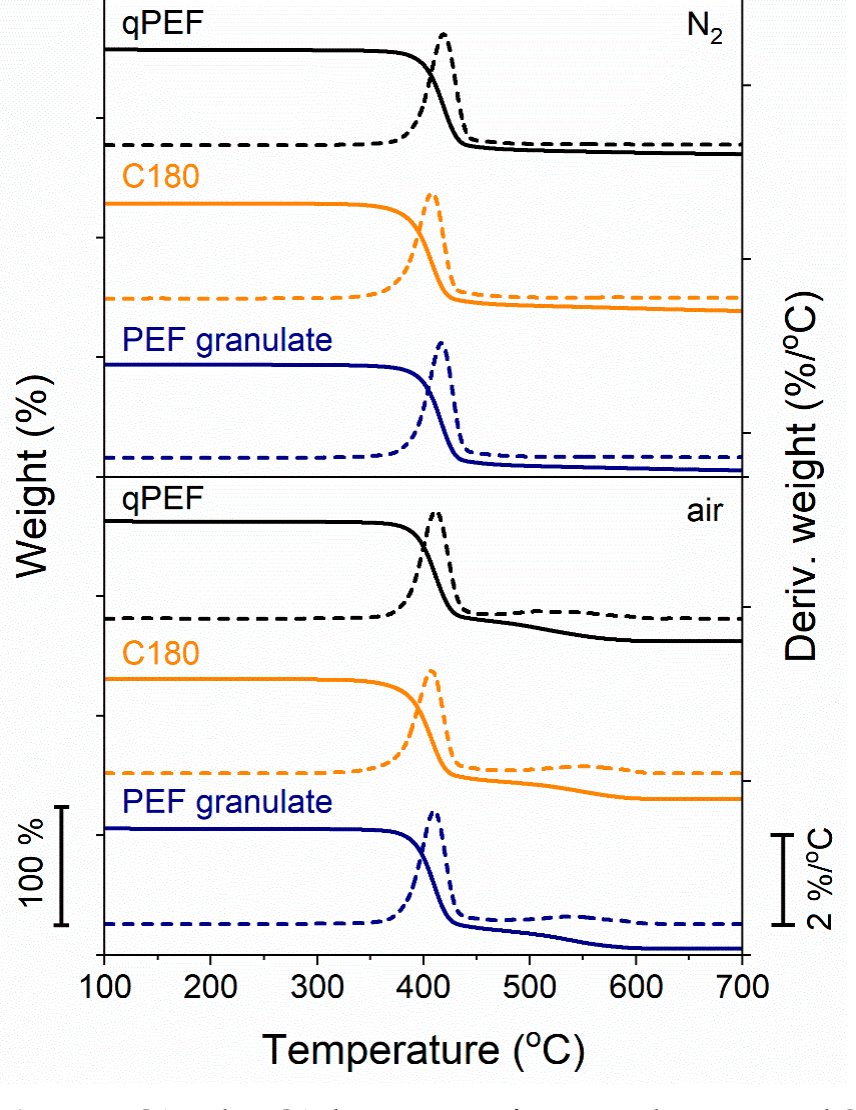
TGA and DTGA thermograms of PEF granulate, qPEF, and C180 recorded during heating at 20 °C/min in a nitrogen atmosphere and in air.

The TGA and DTGA thermograms recorded for selected samples, qPEF, C180, and PEF granulate, in a nitrogen atmosphere and in air are shown in Figure 6, respectively. *T*_5%_ and *T*_d_ are listed in Table 2.

These characteristic temperatures were practically the same for PEF granulate and qPEF, *T*_5%_ of 384–386 °C and *T*_d_ of 417–419 °C in a nitrogen atmosphere, and somewhat lower, 380 °C and 410–411 °C, respectively, in air. This is in agreement with the results of measurements of [*η*], as it was the same, 0.84 dL/g, for PEF granulate and qPEF. In turn, *T*_5%_ of C180 were lower by 13–14 °C than those of qPEF. Also, *T*_d_ of C180 was lower, by approx. 8 °C and 4 °C in a nitrogen atmosphere and in air, respectively. It is worth noting that DTGA curves recorded in air, in addition to the main peaks, exhibited small peaks at 400–550 °C, related to carbonization [43]. According to [44,45], the decomposition of PEF in a nitrogen atmosphere occurs mainly due to β-hydrogen bond scission and to some extent with α-hydrogen bond scission. Others found that the thermal stability of PEF both in a nitrogen atmosphere and in air could be somewhat improved by adding appropriate stabilizers [43].

### 3.2. Structure

**Figure 7:**
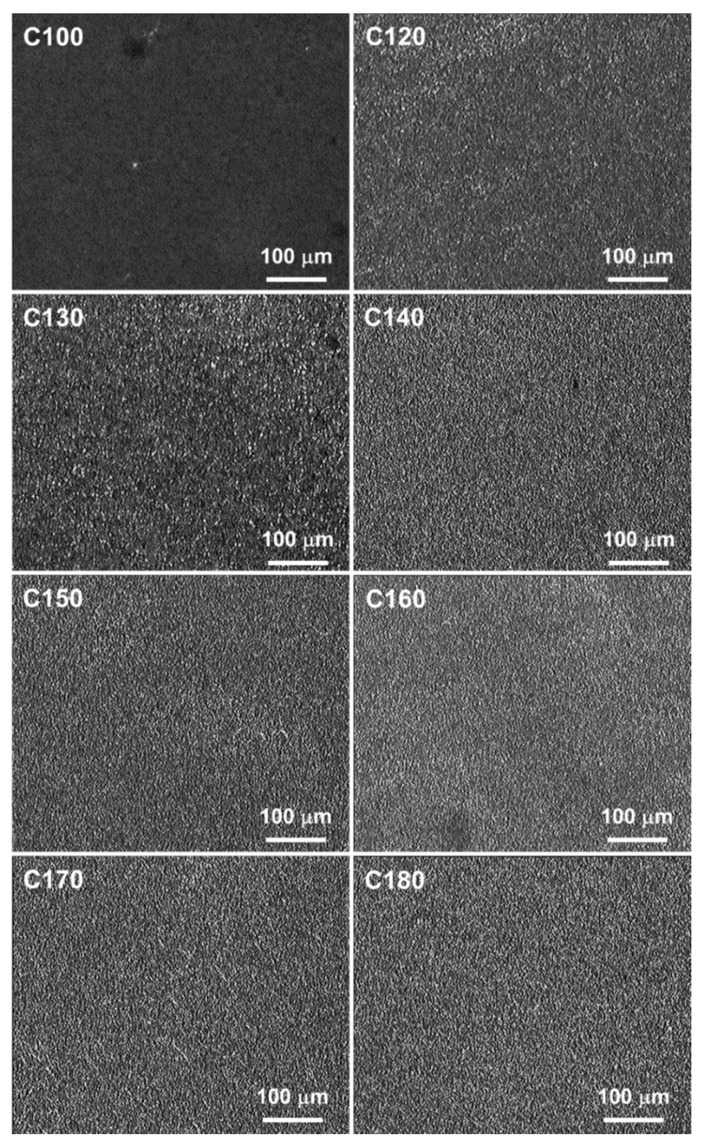
PLM micrographs of thin PEF films cold-crystallized isothermally at different temperatures, *T*_c_. Amorphous C100 sample shown for comparison.

PLM micrographs collected in Figure 7 show the morphologies of PEF samples. PEF held at 100 °C was amorphous, whereas PEF held at 120–180 °C crystallized and contained fine crystalline aggregates, as shown in Figure 7. 

**Figure 8:**
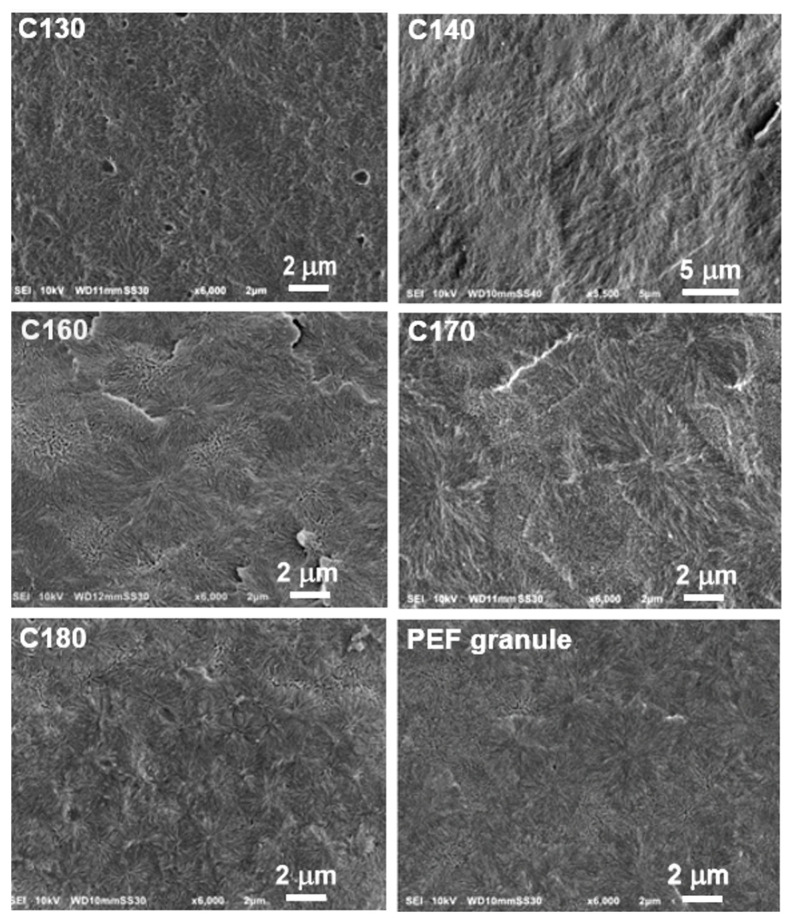
SEM images of etched cryofracture surfaces of PEF cold-crystallized isothermally at different temperatures, *T*_c_, and PEF granule for comparison.

SEM images of etched fracture surfaces of PEF specimens are collected in Figure 8. Etching preferentially removes the amorphous phase and exposes the crystalline phase. The spherulites with centers and radial arrangements of lamellae and also interspherulitic boundaries can be distinguished in all micrographs. Interestingly, the morphology of the PEF granules was also spherulitic. The diameter of spherulites seen in the SEM images varied from about 4 µm to 10 µm and weakly depended on *T*_c_. It is worth noting that a stronger dependence of spherulite size on *T*_c_ was observed for poly(propylene 2,5-furandicarboxylate) (PPE) [46], PET, and poly(ethylene 2,6-naphthalate) (PEN) [47]. 

**Figure 9:**
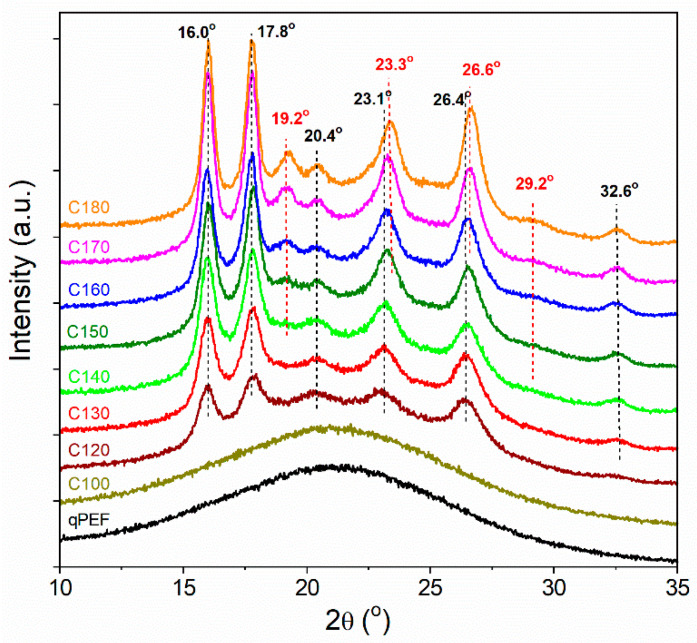
WAXS curves of qPEF, C100 and PEF cold-crystallized at different temperatures *T*_c_. WAXS curves shifted vertically for clarity.

WAXS curves of PEF samples and PEF granule are collected in Figure 9 and Figure S7, respectively. The curves of amorphous qPEF and also C100 show broad halos with maxima at 2*θ* of 21° and approx. 45°, as observed by others [23]. WAXS curves of crystalline samples show several peaks, whose intensity increased with the increasing *T*_c_, especially in the *T*_c_ range from 120 °C to 150 °C. The peaks at 2*θ* of 16.0°, 17.8°, 23.1°, and 26.5° were stronger, whereas those at 20.4° and 32.6° were weak. The WAXS curves of PEF crystallized at *T*_c_ from 160 °C to 180 °C exhibited similar strong peaks at 16.0°, 17.8°, and weak ones at 20.4° and 32.6°. However, in this case, the peaks close to 23° and 26° were shifted by about 0.10–0.15° to the higher 2*θ*, i.e., to 23.3° and 26.6°, respectively. Furthermore, a new peak appeared at 19.2° and a weaker one at 29.2°. These results indicate the presence of different crystallographic modifications in PEF crystallized at low *T*_c_ ≤ 150 °C and high *T*_c_ ≥ 160 °C, which is consistent with the literature data. According to Stoclet et al. [20], the less ordered crystallographic α’-modification formed at lower *T*_c_, and the more ordered α-modification phase formed at higher *T*_c_, i.e., below and above a certain critical temperature. It is worth noting that others found the more ordered crystalline α-form to grow only above 150 °C [24], above 160 °C [20], or above 190 °C [25]. A similar dependence of crystallographic modification on *T*_c_ was observed for PLA, in which the disorder α’-phase and the order α-phase were formed at *T*_c_ < 100 °C and *T*_c_ ≥ 120 °C, respectively [48]. In turn, the diffractogram of the powdered PEF granule was typical of the α’-phase (Figure S7). It is worth mentioning that in the WAXS curves of analyzed materials, no peaks typical of the β-form at approx. 9.5° were found [25]. Recently, Maini et al. [23] proposed a triclinic unit cell for the α’-form and a monoclinic unit cell for the α- and β-forms based on the best fitting of X-ray diffraction powder patterns. 

**Figure 10:**
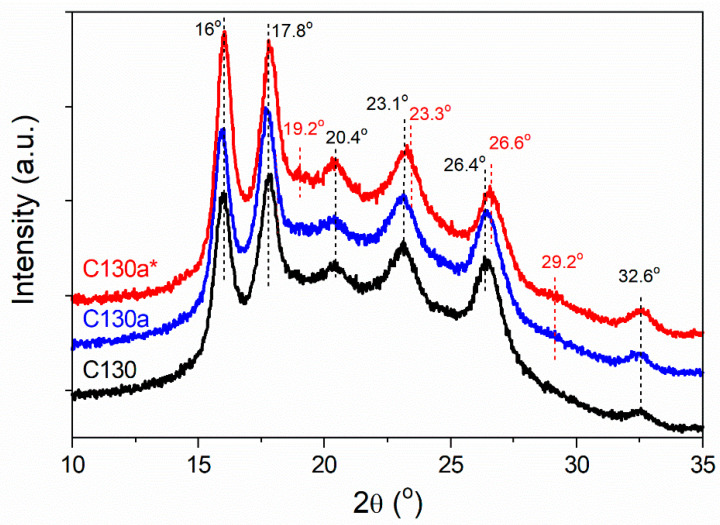
Comparison of WAXS curves of C130*,* re-heated C130a and C130a* held at *T*_a_ = 170 °C for 15 s and 1 h, respectively, and then quickly quenched to RT. WAXS curves shifted vertically for clarity.

The influence of re-heating on the crystalline structure is illustrated in Figure 10, where the WAXS curves of C130, C130a, and C130a* (held at 170 °C for 1 h) are compared. The reorganization of the structure in C130a and 130a* was confirmed by DSC thermograms by a shift of the *T*_m1_ peak to a higher temperature as shown in Figure S8.

It follows that C130a contained the α’-form, whereas in C130a* the α-phase was present, as is reflected in the increase in scattering intensity at 2*θ* of 19.2° and 29.2° as well as by a shift of the peaks near 23° and 26 to higher 2*θ* angles. This suggests the α-phase formation in C130a* at 170 °C. This effect was less pronounced after re-heating of C140 and C150 with initially higher *X*_c_, and especially C160 already containing the α-form, despite higher *T*_a_, as shown in Figure S9. It should be mentioned that the α’ to α-form transformation induced by heating and the coexistence of both forms were observed by others [24]. 

**Figure 11:**
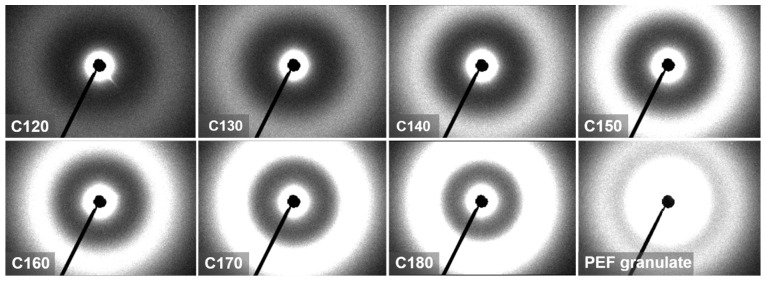
SAXS-2D patterns of PEF cold-crystallized isothermally at temperatures, *T*_c_, ranging from 120 °C to 180 °C, and PEF powdered granules for comparison.

Figure 11 shows SAXS-2D isotropic patterns of PEF cold-crystallized at different *T*_c_ and powdered PEF granules for comparison. The latter shows a significant contribution of scattering at small 2*θ* angles. The scattering intensity distributions vs. 2*θ* of the cold-crystallized PEF showed pronounced maxima. With increasing *T*_c_ the intensities of the maxima increased and shifted to smaller 2*θ* angles and this effect was stronger for the re-heated samples, as shown in Figure S10.

**Figure 12:**
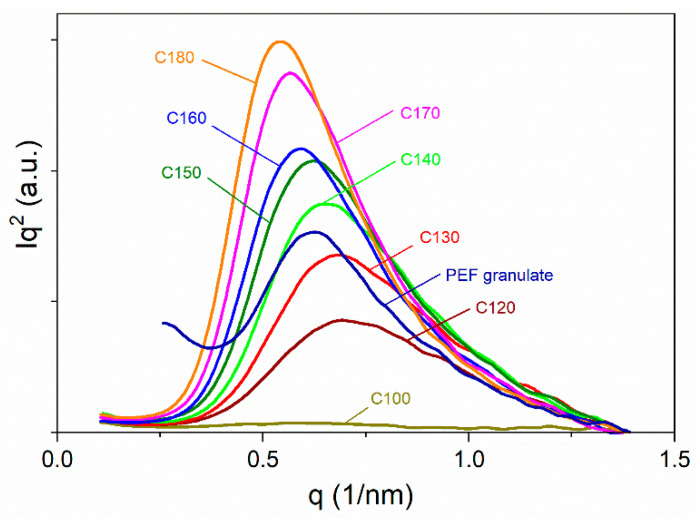
Kratky plots, *Iq*^2^ vs. *q*, where: *I* is scattering intensity; and q=4π sin⁡θ/λ, *λ* is wavelength, for cold-crystallized PEF and PEF granulate. Plot for C100 shown for comparison.

The Kratky plots corresponding to SAXS-2D patterns in Figure 11 are collected in Figure 12, whereas those of the re-heated samples are in Figure S11. An increase in the scattering intensity and the shift of the peaks to lower *q* values with the increasing *T*_c_, and also due to re-heating, were observed. The mean long period (*LP*) values, calculated based on the peak positions according to the Bragg law, are listed in Table 3 together with *LP* and average lamella thickness (*L*_c_) based on 1D-correlation functions *F(x)* [49]. The *F(x)* functions for all crystalline samples are plotted in Figure S12.

As follows from the data in Table 3, the *LP* of the lamellar structure increased slightly with the increasing *T*_c_, from 8.9 nm and 7.7 nm for C120 to 11.4 and 10.6 nm for C180. The *L*_c_ also increased, from 3.0 nm to 3.5 nm. The *L*_c_ values are close to the 4 nm *L*_c_ value of PEF crystallized at 165 °C, which was reported by Mao et al. [50]. The *LP* and *L*_c_ of the re-heated samples are slightly larger. The small increase in *L*_c_ with the increasing *T*_c_ could contribute to the weak dependence of *T*_m2_ on *T*_c_. No evidence of two different long periods was found, which suggests that the less stable crystals with *T*_m1_ were located between those with *T*_m2_. Taking into account the predominance of the crystals with higher melting temperature *T*_m2_, it can be concluded the *L*_c_ and *LP* vales are related to this crystal population.

## 4. Conclusions

The effect of *T*_c_ on the morphology and thermal properties of the cold-crystallized PEF was studied. Melt-quenching of PEF resulted in an amorphous state, and this amorphous polymer crystallized only at temperatures well above 100 °C. PEF was isothermally cold-crystallized at selected temperatures (*T*_c_) in the range of 120 °C to 180 °C. In PEF crystallized at *T*_c_ ≤ 150 °C, the less ordered crystallographic α’-phase was formed, whereas at higher temperatures the polymer crystallized in the α-form, as evidenced by WAXS. The mean long period of 8.9–11.4 nm and 7.7–10.6 nm, and small lamella thickness of 3.0 to 3.5 nm, determined by SAXS, only weakly depended on *T*_c_. 

The analysis of the etched surfaces revealed the spherulitic morphology of PEF. The diameter of the spherulites ranged from about 4 µm to 10 µm and weakly depended on *T*_c_.

Crystallinity (*X*_c_) increased with increasing *T*_c_ from approx. 29% at 120 °C to approx. 41% at 180 °C. A similar high value of 41% was detected for the PEF as received; however, its crystalline phase was in the α’-form. Based on the three-phase model, the content of the mobile amorphous phase (*X*_maf_) and the rigid amorphous phase (*X*_raf_) was determined. *X*_raf_ decreased with the increased *T*_c_ as at a higher temperature polymer chain mobility is enhanced, which facilitates the organization of the segments into ordered crystalline structures, with reduced stress transmitted to the amorphous segments and a lower fraction of chain segments subjected to geometrical constraints. 

The melting behavior of PEF was complex. DSC thermograms exhibited low-temperature melting peaks centered at *T*_m1_, which increased linearly with *T*_c_, and the main ones with higher *T*_m2_ were nearly independent of *T*_c_. At *T*_c_ of 170 °C and 180 °C, the low-temperature peaks turned into shoulders on ascending slopes of the main peaks. The low-temperature peaks resulting from the melting of less stable crystals were observed already after short crystallization at *T*_c_, although their *T*_m1_ values were lower than those after longer crystallization at the same *T*_c_. This evidenced the formation of the less stable crystals at the early stages of crystallization and their isothermal annealing, perfectioning, and/or thickening during further crystallization at *T*_c_. The content of these less stable crystals in the crystalline phase did not exceed 20%. Re-heating the cold-crystallized PEF to the temperature above the low-temperature melting peak followed by cooling resulted in recrystallization of the less stable crystals into a more stable phase melting at a higher temperature. The effect of this treatment on the structure was also investigated.

The cold-crystallization conditions and the elaborated re-heating protocol controlled the structure and thermal properties of the biobased PEF. Low *T*_c_ resulted in the α’-phase formation having less crystallinity and a higher content of the rigid amorphous phase. At high *T*_c_, the α-phase formed, higher crystallinity developed, the rigid amorphous phase content was lower, and the melting of less stable crystals occurred at a higher temperature; however, slight polymer degradation could occur. The applied thermal treatment altered the thermal behavior of PEF by shifting the melting of the less stable crystals to a significantly higher temperature.

## Figures and Tables

**Table 1:** Calorimetric parameters of cold-crystallized PEF determined from DSC thermograms recorded at 10 °C/min. *T*_g_—glass transition temperature; *T*_s_—temperature of shoulder on ascending slope of main melting peak; *T*_m_ and Δ*H*_m_—melting peak temperature and melting enthalpy; Δ*H*_mc_—total melting enthalpy; *X*_c_—degree of crystallinity calculated assuming the heat of fusion of crystals of 137 J/g [3]. Asterisks denote approximate values. Double asterisks indicate that from Δ*H*_mc_ the small exothermic effect enthalpy was subtracted: 1.5 J/g for C120, 0.2 J/g for C130.

Sample	*T*_g_ (°C)	*T*_m1_ (°C)	Δ*H*_m1_ (J/g)	*T*_s_ (°C)	*T*_m2_ (°C)	Δ*H*_m2_ (J/g)	Δ*H*_mc_ (J/g)	*X*_c_ (%)
C120	89	141	5.8	192 *	211	35.3	39.3 **	28.7
C130	88	150	7.0	193 *	210	38.0	44.9 **	32.8
C140	88	160	8.8	197 *	210	37.2	46.0	33.6
C150	89	168	10.1 *	200 *	209	38.8	48.9	35.7
C160	88	179	11.9 *	-	205	40.8 *	52.7	38.6
C170	88	191	-	-	207	-	54.4	39.7
C180	88	203 *	-	-	212	-	56.3	40.7
PEF	90	-	-	-	213	-	55.5	40.5

**Table 2:** Thermogravimetric parameters of PEF specimens measured during heating at 20 °C/min in a nitrogen atmosphere and in air. *T*_5%_ denotes 5% weight loss temperature whereas *T*_d_ is DTGA peak temperature.

Sample	Nitrogen		Air		
	*T*_5%_ (°C)	*T*_d_ (°C)	*T*_5%_ (°C)	*T*_d1_ (°C)	*T*_d2_ (°C)
PEF granulate	384	417	380	410	540
qPEF	386	419	380	411	512
C180	370	408	367	407	550

**Table 3:** Parameters of lamellar structure of PEF samples. *LP*(K)—mean long period determined based on Kratky plots, *LP*(c) and *L*_c_(c)—mean long period and lamella thickness, respectively, determined based on 1D correlation function.

Sample	*LP*(K) (nm)	*LP*(c) (nm)	*L*_c_(c) (nm)
C120	8.9	7.7	3.0
C130	9.1	8.1	3.0
C130a	10.1	9.6	3.1
C140	9.5	8.6	3.1
C140a	10.5	9.8	3.2
C150	10.0	9.5	3.2
C150a	11.2	10.6	3.3
C160	10.6	10.0	3.3
C160a	11.2	10.5	3.4
C170	11.1	10.2	3.4
C180	11.4	10.6	3.5
PEF granulate	9.9	9.6	3.3

## Data Availability

The original contributions presented in the study are included in the article, further inquiries can be directed to the corresponding author.

## Supplementary Materials

The following supporting information can be downloaded at: https://www.mdpi.com/article/10.3390/polym16213052/s1. Figure S1: repeating unit of PEF. Figure S2: ATR-FTIR spectrum of amorphous PEF. Figure S3: DSC thermograms recorded during isothermal cold-crystallization at 180 °C for 12 h of initially amorphous samples: compression-molded qPEF and PEF granulate. Figure S4: DSC heating thermograms of cold-crystallized PEF recorded at 30 °C/min. Figure S5: Crystallinity, *X*_c_, content of amorphous phase, *X*_a_, and rigid amorphous fractions, *X*_raf_, vs. cold-crystallization temperature, *T*_c_. Figure S6: Temperature dependencies of storage and loss moduli of qPEF and C160 samples. Figure S7: WAXS curve recorded for powdered PEF granulate. Figure S8: Comparison of DSC heating thermograms of cold-crystallized sample C130 and its re-heated-at-*T*_a_ = 170 °C counterparts C130a (ta = 15 sec) and C130a* (ta = 1 h) recorded at 10 °C/min. Figure S9: Comparison of WAXS curves of PEF samples cold-crystallized isothermally at 140 °C, 150 °C, and 160 °C with those of corresponding re-heated samples. Figure S10: SAXS intensity distributions vs. scattering angle 2θ for isothermally cold-crystallized PEF and PEF granulate as received (a), and comparison of the scattered intensity distributions for isothermally cold-crystallized PEF samples before and after the re-heating (b). Figure S11: Kratky plots *Iq*^2^ vs. *q*, where *I* is scattering intensity, q=4π sin⁡θ/λ, λ is wavelength, calculated based on SAXS intensity distribution for isothermally cold-crystallized PEF samples before and after re-heating. Figure S12: 1D correlation function, F(x), for isothermally cold-crystallized PEF samples before and after re-heating, and for PEF powdered granulate.
