# Supplementary material for: Crystallization-Controlled Structure and Thermal Properties of Biobased Poly(Ethylene2,5-Furandicarboxylate)
Source: Polymers (Basel). 2024 Oct 30;16(21):3052. doi: 10.3390/polym16213052 (PMC11548705; doi:10.3390/polym16213052)
Supplement: Supplementary file 1 [file polymers-16-03052-s001.zip › polymers-3221089-supplementary.pdf]

Supplementary Materials:

Crystallization-Controlled Structure and Thermal Properties of Biobased Poly(Ethylene2,5-Furandicarboxylate)

Miroslaw Pluta [1,*], Joanna Bojda [1], Mariia Svyntkivska [1], Tomasz Makowski [1,*], Ele L. de Boer [2] and Ewa Piorkowska [1]

[1] Centre of Molecular and Macromolecular Studies, Polish Academy of Sciences, Sienkiewicza 112, 90-363 Lodz, Poland
[2] Avantium Renewable Polymers BV, Zekeringstraat 29, 1014 BV Amsterdam, The Netherlands
* Correspondence: miroslaw.pluta@cbmm.lodz.pl (M.P.); tomasz.makowski@cbmm.lodz.pl (T.M.)

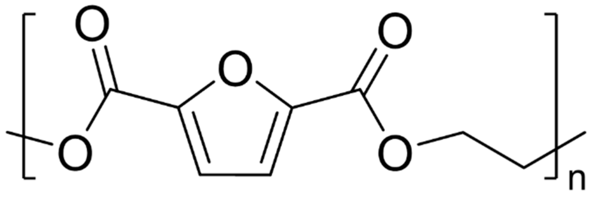

Figure S1. Repeating unit of PEF.

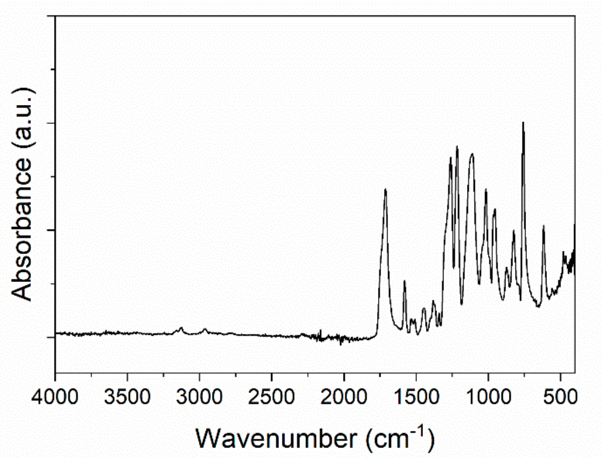

Figure S2. ATR-FTIR spectrum of amorphous PEF.

Preliminary tests were focused on determining the time required for the accomplishment of isothermal cold-crystallization at the highest crystallization temperature (T_c) of 180 °C of qPEF and PEF granule after melting and quenching in DSC. Isothermal crystallization thermograms of these samples (Figure S3) evidenced the long crystallization lasting nearly 12 h. Based on this experiment, the time required to accomplish the cold crystallization was determined as 12 h or more.

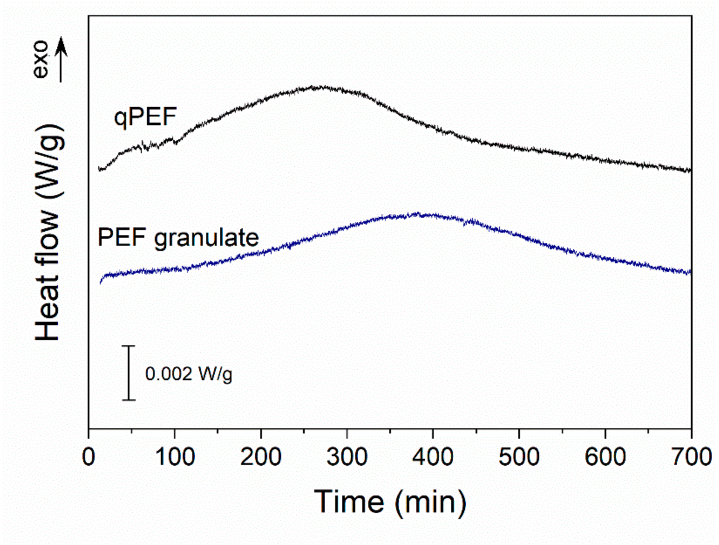

Figure S3. DSC thermograms recorded during isothermal cold-crystallization at 180 °C for 12 h of initially amorphous samples: compression molded qPEF and PEF granulate.

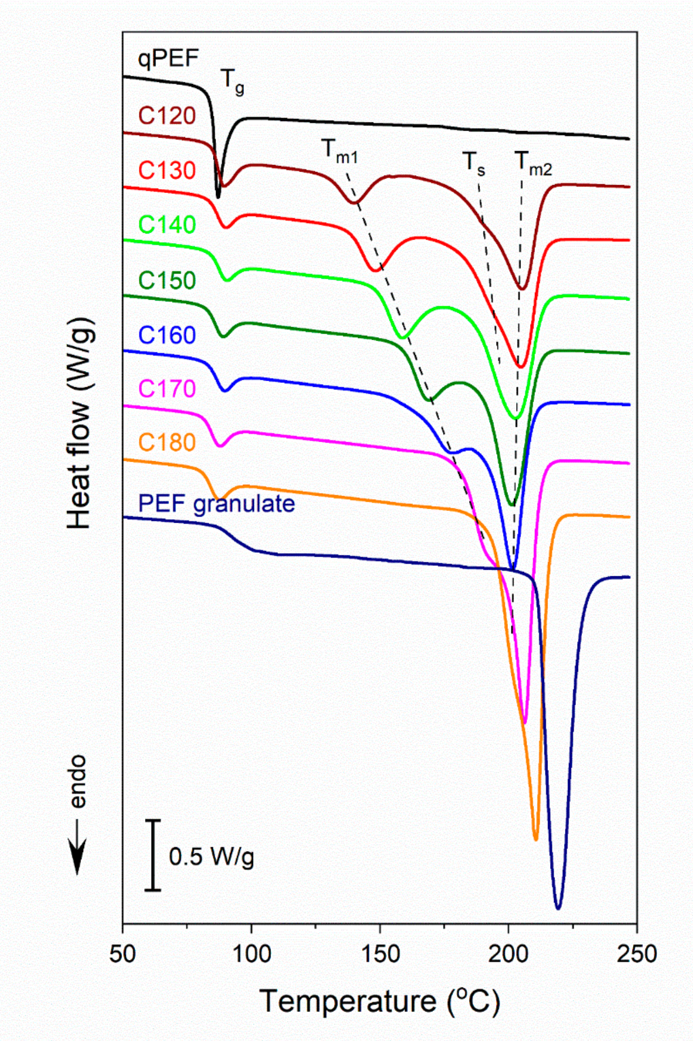

Figure S4. DSC heating thermograms of cold-crystallized PEF recorded at 30 °C/min. Thermograms shifted vertically for clarity.

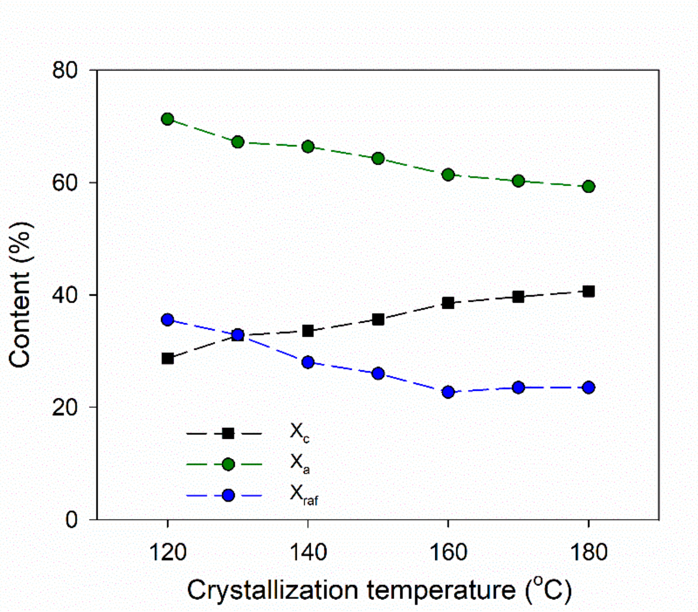

Figure S5. Crystallinity, X_c, content of amorphous phase, X_a, and rigid amorphous fractions, X_{raf}, vs. cold-crystallization temperature, T_c.

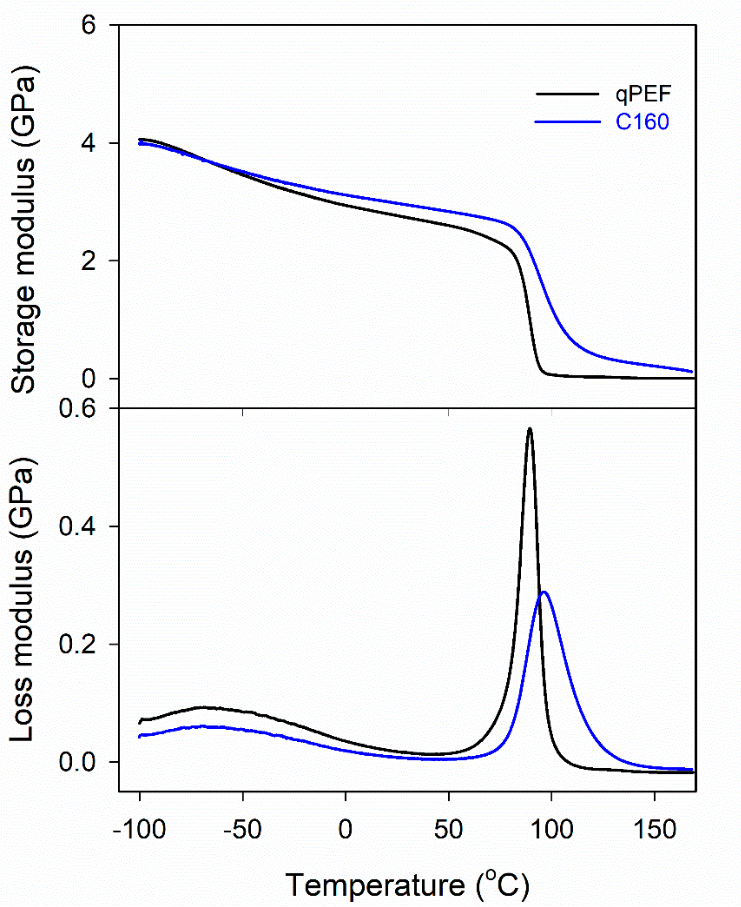

Figure S6. Temperature dependencies of storage and loss moduli of qPEF and C160 samples.

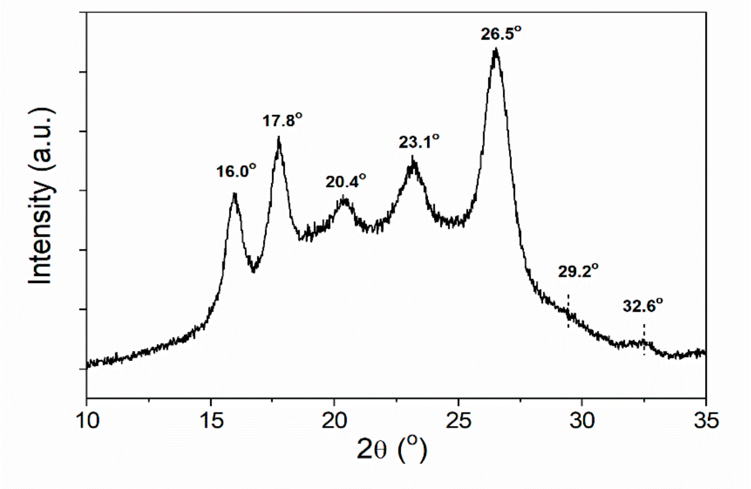

Figure S7. WAXS curve recorded for powdered PEF granulate.

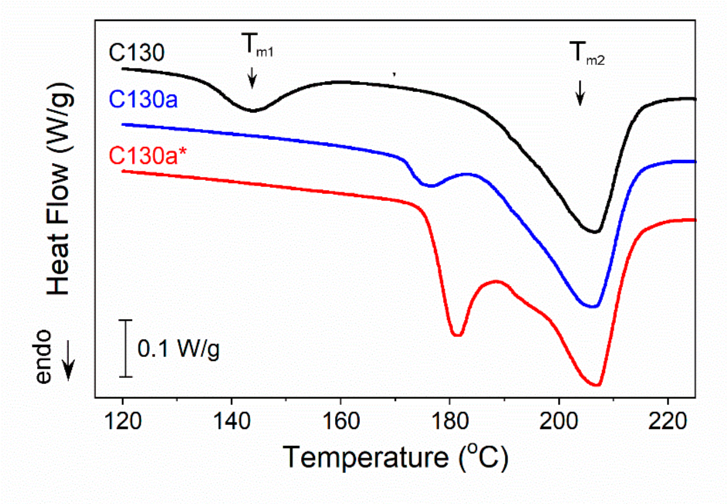

Figure S8. Comparison of DSC heating thermograms of codl-crystallized sample C130 and its re-heated at T_a=170 °C counterparts C130a (ta=15 sec) and C130a* (ta=1h) recorded at 10 °C/min.

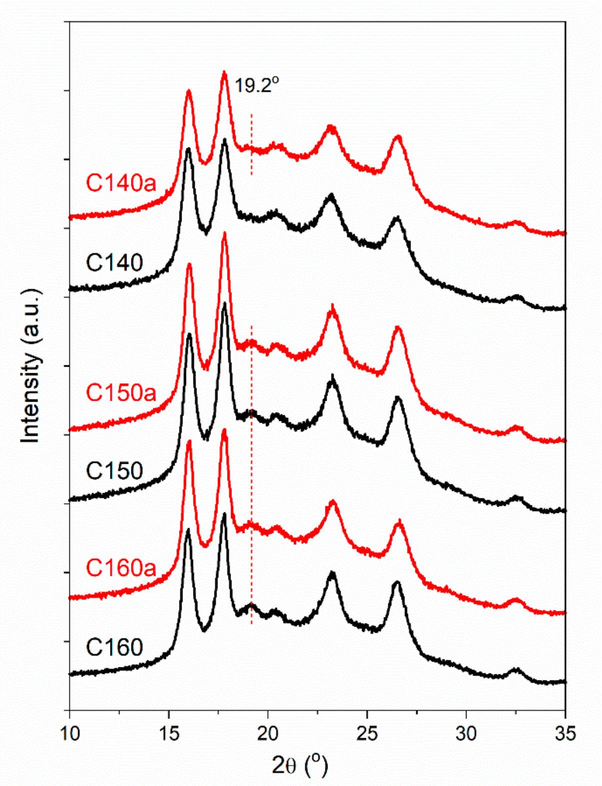

Figure S9. Comparison of WAXS curves of PEF samples cold-crystallized isothermally at: 140 °C, 150 °C and 160 °C, with those of corresponding re-heated samples.

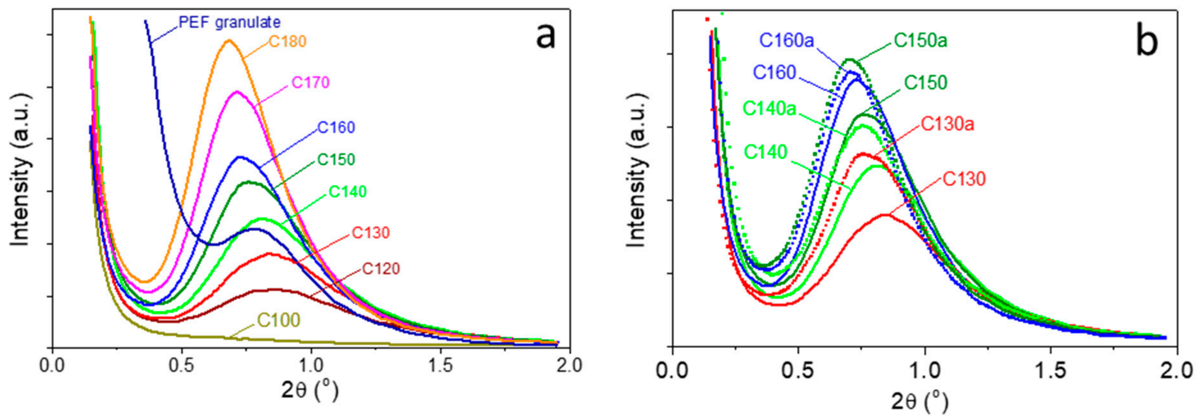

Figure S10. SAXS intensity distributions vs. scattering angle 2θ for isothermally cold-crystallized PEF and PEF granulate as received (a), and comparison of the scattered intensity distributions for isothermally cold-crystallized PEF samples before and after the re-heating (b).

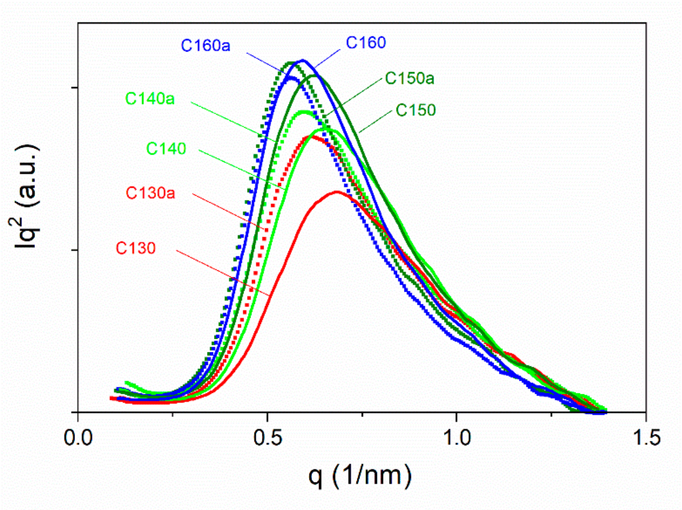

Figure S11. Kratky plots Iq^2 vs. q where: I is scattering intensity, $q = 4\pi \sin\theta/\lambda$, λ is wavelength, calculated based on SAXS intensity distribution for isothermally cold-crystallized PEF samples before and after re-heating.

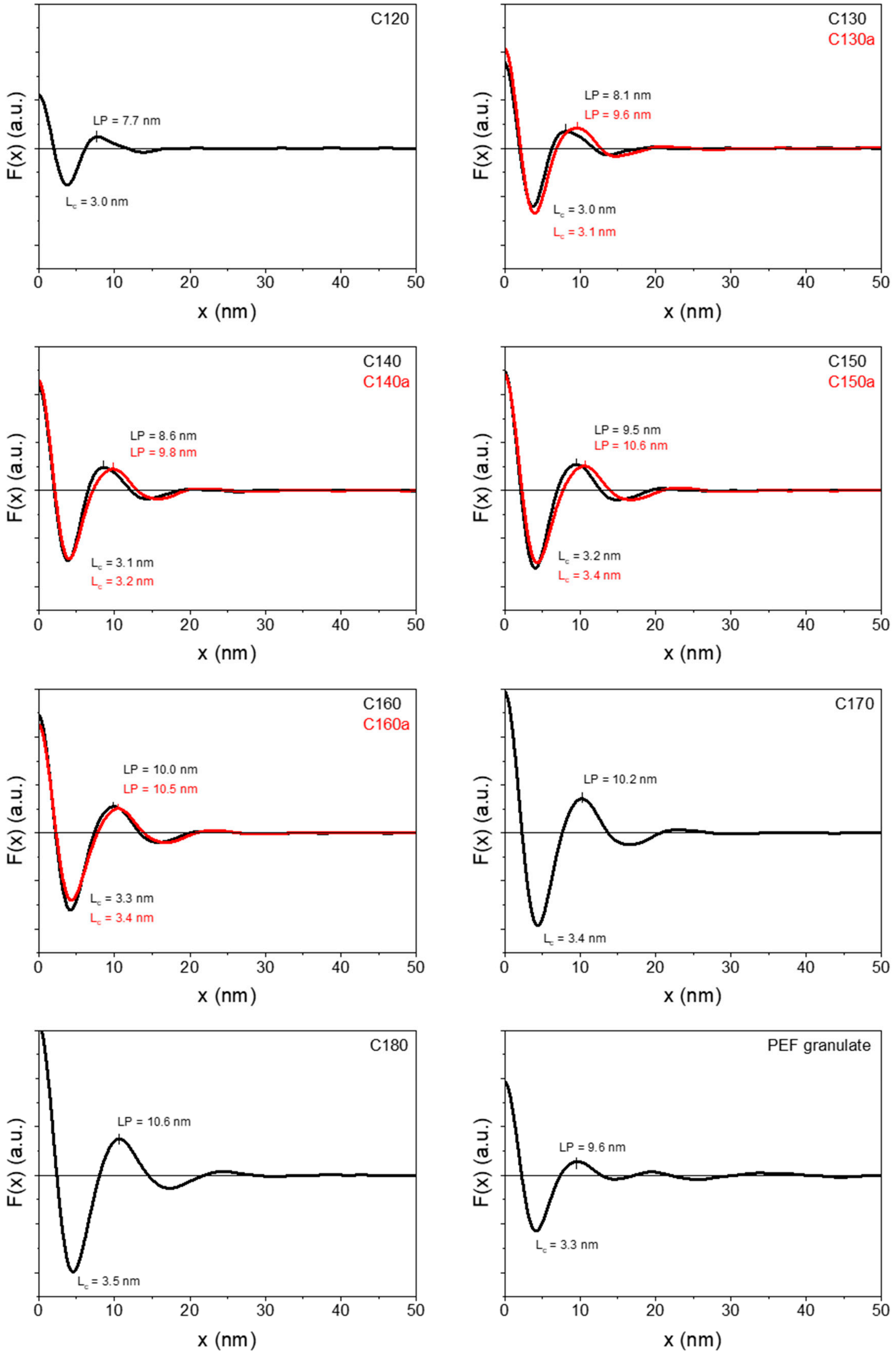

Figure S12. 1D correlation function, F(x), for isothermally cold-crystallized PEF samples before and after re-heating, and for PEF powdered granulate.

The position of the first maximum corresponds to the mean long period, LP(c). The intersection of extrapolation of the linear fragment with the tangent to the first minimum corresponds to the mean lamella thickness.
